# Late-onset dyshormonogenic goitrous hypothyroidism due to a homozygous mutation of the *SLC26A7* gene: a case report

**DOI:** 10.1186/s13052-024-01672-3

**Published:** 2024-05-29

**Authors:** Elisabetta Sciarroni, Lucia Montanelli, Caterina Di Cosmo, Brunella Bagattini, Simone Comi, Luisa Pignata, Alessandro Brancatella, Giuseppina De Marco, Eleonora Ferrarini, Chiara Nencetti, Maria Rita Sessa, Francesco Latrofa, Ferruccio Santini, Massimo Tonacchera, Patrizia Agretti

**Affiliations:** 1 Department of Clinical and Experimental Medicine, Endocrine Unit, Research Center of Excellence AmbiSEN, University of Pisa, 56124 Pisa, Italy; 2 Laboratory of Chemistry and Endocrinology, University Hospital of Pisa, 56124 Pisa, Italy

**Keywords:** *SLC26A7* gene, Congenital hypothyroidism, Dyshormonogenic goiter, Genetic analysis, Case report

## Abstract

**Background:**

In this study, we used targeted next-generation sequencing (NGS) to investigate the genetic basis of congenital hypothyroidism (CH) in a 19-year-old Tunisian man who presented with severe hypothyroidism and goiter.

**Case presentation:**

The propositus reported the appearance of goiter when he was 18. Importantly, he did not show signs of mental retardation, and his growth was proportionate. A partial organification defect was detected through the perchlorate-induced iodide discharge test. NGS identified a novel homozygous mutation in exon 18 of the *SLC26A7* gene (P628Qfs*11), which encodes for a new iodide transporter. This variant is predicted to result in a truncated protein. Notably, the patient's euthyroid brother was heterozygous for the same mutation. No renal acid–base abnormalities were found and the administration of 1 mg of iodine failed to correct hypothyroidism.

**Conclusions:**

We described the first case of goitrous CH due to a homozygous mutation of the *SLC26A7* gene diagnosed during late adolescence.

## Background

Congenital hypothyroidism (CH) may result from thyroid dysgenesis (80%) or dyshormonogenesis (20%), the latter due to mutations in genes involved in thyroid hormone synthesis, namely *NIS*, *DUOX2*, *DUOXA2*, *TG*, *TPO*, *SLC26A4*, and *DEHAL1* [1–3]. SLC26A7, also known as a Cl-/HCO3- exchanger in the kidney [4–7], is part of the SLC26 transporter family, which includes multiple anion exchangers such as pendrin, with an affinity for iodide and chloride. This molecule has been identified as a new iodide transporter expressed at the apical membrane of thyroid follicular cells, playing a significant role in thyroid hormone biosynthesis. Recent exome sequencing studies have implicated *SLC26A7* gene mutations as a new cause of CH [8–10]. In the study, we used targeted Next Generation Sequencing (NGS) to investigate a young man with goiter and hypothyroidism. This led to the discovery of a novel homozygous mutation in exon 18 of the *SLC26A7* gene (P628Qfs*11), predicted to translate into a truncated protein.

## Case presentation

**Fig. 1:**
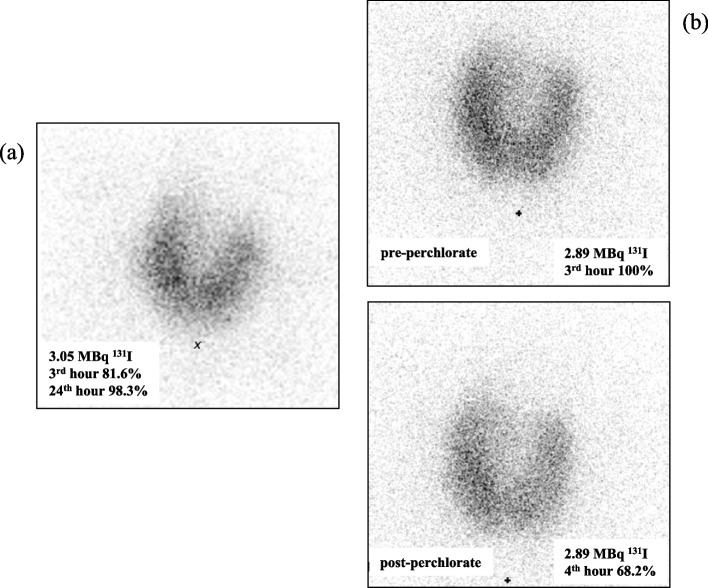
Thyroid scintigraphy with 131I (**a**) and perchlorate discharge test (**b**)

The patient was a 19-year-old Tunisian man who moved to Italy in 2021. Two months after his arrival, he experienced symptomatic neck enlargement, which was confirmed as a diffuse thyroid goiter by ultrasound (US). Initial blood tests revealed severe primary hypothyroidism (FT4 0.06 ng/dL n.v. 0.90–1.70, FT3 0.50 pg/mL n.v 2.10–4.30, TSH 396.80 µIU/mL n.v. 0.27–4.20) and the patient was promptly referred to our Center for further investigations. Notably, he exhibited no signs of mental retardation or explicit hypothyroidism symptoms. He reported no personal or familial history of thyroid disease or medication use. At the time of admission, his height was 1,76 m, his weight 62,5 kg, and his body mass index 20,2 kg/m^2^, indicative of normal harmonious growth. Physical examination showed a myxedematous face, with periorbital and labial edema, and neck examination identified a large diffuse goiter. No other signs of hypothyroidism, such as macroglossia, umbilical hernia or cutaneous annexes abnormalities were detected. The patient presented with severe primary hypothyroidism, as indicated by thyroid function tests (Table 1), and high levels of transaminases and lactate dehydrogenase, compatible with the state of hypothyroidism (data not shown). Ultrasound confirmed a diffuse goiter (thyroid volume 94 ml). A tru-cut biopsy showed a micro-macrofollicular thyroid with hyperplastic features. Autoimmune and infiltrative thyroid diseases were excluded, and no iatrogenic/toxic implication was detected. Thyroid scintigraphy with 131I showed increased uptake (81.6% and 98.3% after 3 and 24 h, respectively) (Fig. 1a) and the perchlorate-induced iodide discharge test indicated a 31.8% discharge rate (Fig. 1b), suggesting a partial organification defect. L-thyroxine (LT4) replacement therapy was initiated and titrated, resulting in euthyroidism and goiter reduction (thyroid volume 41 ml). Urinary parameters were also evaluated to assess potential kidney-related fluids and electrolytes alterations, but no significant abnormalities were observed (Table 2). The patient’s brother, the only relative available for examination, was clinically and biochemically euthyroid (FT4 1.06 ng/dL; FT3 5.16 pg/mL; TSH 1.1 mIU/mL; AbTPO and AbTg undetectable).

**Table 1:** Patient’s serum thyroid function tests at the time of admission in our hospital

Thyroid Parameter	Value	Reference Range
FT4 (ng/dL)	< 0.1	0.7 – 1.7
FT3 (ng/L)	1.73	2.7 – 5.7
rT3 (ng/mL)	0,077	0,069 – 0,262
TSH (mIU/L)	348	0.4 – 4
Tg (ng/mL)	3724.68	< 35
AbTg (IU/mL)	< 1	< 30
AbTPO (IU/mL)	< 1	< 35

*Abbreviations: FT4* Free thyroxine, *FT3* Free triiodothyronine, *rT3* Reverse free triiodothyronine, *TSH* Thyroid stimulating hormone, *Tg* Thyreoglobulin, *TgAb* Anti-thyroglobulin antibodies, *AbTPO* Antithyroid peroxidase antibodies

**Table 2:** Patient’s urinary parameters

Urinary Parameter	Value	Reference Range
pH	6.0	5.5 – 7.5
Creatinine (mg/24 h)	1760	980—2200
Sodium (mEq/24 h)	154	40—220
Potassium (mEq/24 h)	46	25—125
Calcium (mg/24 h)	110	100—300
Magnesium (mg/24 h)	130	72.9 – 121.5
Albumin (mg/24 h)	4	< 30
*Diuresis 1000 ml*		

Patient’s urinary parameters evaluated to assess potential kidney-related fluids and electrolytes alterations associated a posteriori with the SLC26A7 gene mutation

Thyroid morphology and function were evaluated using thyroid ultrasound, perchlorate discharge test as well as measurements of serum FT4, FT3, TSH, anti-TG and anti-TPO antibodies, as described [11]. A twenty-four-hour urine collection was performed to determine urine ionic composition via potentiometric system. Written informed consent for genetic analyses and for the scientific use of data were obtained from the proband and his brother in accordance with the Declaration of Helsinki and subsequent amendments (Good Clinical Practice guidelines).

Targeted NGS was conducted to investigate the cause of the goitrous hypothyroidism in our patient. We designed a custom panel targeting 34 genes involved in primary CH pathogenesis [10, 12–16] (Table 3). Genomic DNA was isolated from peripheral blood cells. For library preparation, we used the SureSelect QXT Reagent Kit (Agilent Technologies Inc., Santa Clara, CA, USA). Custom capture probes were then hybridized to the target sequences of the library for sequence enrichment. The enriched library was amplified using dual indexing primers. Equimolar amounts of multiple libraries were pooled into a single sample and sequenced on an Illumina MiSeq Dx System platform (Illumina Inc, San Diego, CA, USA) using the MiSeq Reagent Nano Kit V2 300 Cycles (2 × 151 bp paired-end run). The MiSeq platform generated a pair of FastQ files per sample suitable for secondary analysis with SureCall NGS software version 4.2.1 (Agilent Technologies Inc., Santa Clara, CA, USA). We performed in silico analysis of clinically relevant variants using the free web-based softwares Mutation Taster, PROVEAN and MutPred to estimate the variant's impact on the gene product. The selected variants were validated by Sanger sequencing [17]. Segregation analysis was subsequently performed.

**Table 3:** Custom panel targeting 34 genes involved in primary CH pathogenesis

Gene	Region	NCBI RefSeq
*ASXL3*	chr18: 31158173–31331169	NM_030632.3
*CDCA8*	chr1: 38158080–38175401	NM_018101
*DIO1*	chr1: 54356902–54376769	NM_000792.7
*DIO2*	chr14: 80663859–80854110	NM_000793
*DIO3*	chr14: 102027678–102029799	NM_001362
*DNAJC17*	chr15:41057349–41099686	NM_018163.3
*DUOX1*	chr15: 45422121–45457784	NM_017434
*DUOX2*	chr15: 45384838–45406552	NM_014080
*DUOXA1*	chr15: 45409554–45422146	NM_144565
*DUOXA2*	chr15: 45406509–45410629	NM_207581
*GLIS3*	chr9: 3824117–4348402	NM_152629
*GNAS*	chr20: 57414763–57486261	NM_000516.7
*HHEX*	chr10: 94447935–94455414	NM_002729.5
*HOXB3*	chr17: 46626222–46682284	NM_001330322
*HOXD3*	chr2: 177001330–177037840	NM_006898.5
*IYD*	chr6: 150690018–150727115	NM_203395.3
*JAG1*	chr20: 10618322–10654704	NM_000214.3
*NKX2-5*	chr5: 172659102–172662370	NM_001166175
*NTN1*	chr17: 8924817–9147327	NM_004822.3
*PAX8*	chr2: 113973564–114036537	NM_003466.4
*SECISBP2*	chr9: 91933405–91974588	NM_024077.5
*SLC16A2*	chrX: 73641075–73753762	NM_006517.5
*SLC26A4*	chr7: 107301070–107358264	NM_000441.2
*SLC26A7*	chr8: 92221712–92410393	NM_001282356
*SLC5A5*	chr19: 17982744–18005993	NM_000453.3
*TBX1*	chr22: 19744216–19771126	NM_080647
*TG*	chr8: 133879193–134147157	NM_003235.5
*THRA*	chr17: 38214533–38250130	NM_001190918
*THRB*	chr3: 24158634–24537257	NM_000461
*TPO*	chr2: 1377985–1547493	NM_000547
*TSHR*	chr14: 81421323–81612660	NM_000369.5
*TTF1*	chr14: 36516392-36521149	NM_001079668.3
*TTF2*	chr9: 97853226-97856717	NM_004473.4
*TUBB1*	chr20: 57594299–57601719	NM_030773.4

Name, chromosome localization and NCBI reference transcript of the 34 candidate genes included in NGS panel

**Fig. 2:**
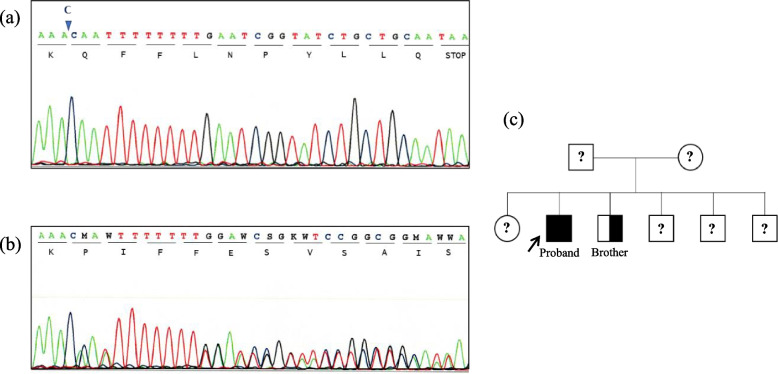
Sanger sequencing and segregation analysis of the pathogenic variant of the SLC26A7 gene. Patient’s sequence electropherogram showing the homozygous deletion of the C (**a**), brother’s sequence electropherogram showing the heterozygous deletion of the C (**b**) and family pedigree (**c**). Empty symbols with a “?” inside represent family members not investigated

SureCall software identified 6 pathogenic gene variants, five of which were UTR or non-coding transcript variants for the IYD and HOXB3 genes. The sixth variant, identified as chr8:92406214 AC > A, was located in the coding region of the *SLC26A7* gene. This variant, registered as dbSNP ID rs768718640, affected transcripts NM_001282356, NM_001282357, NM_052832, and NM_134266, leading to a frameshift mutation with a high impact. Specifically, the homozygous deletion of the C at position 1883 of the exon 18 of the *SLC26A7* gene caused a proline to glutamine substitution at position 628 of the protein, a frameshift, and a formation of a stop codon (TAA) after 11 amino acids. GnomAD Genome indicated a minor allele frequency of 0.00002833 suggesting that the variant is exceptionally rare in the general population. The new variant called c.1883delC or p.P628Qfs*11 was not present in ClinVar and was predicted to be pathogenic by MutationTaster, PROVEAN and MutPred. The Exome Aggregation Consortium accessed in January 2023 did not report homozygous cases (ExAC, https://gnomad.broadinstitute.org/) for this truncating variant. Sanger sequencing confirmed the presence of the homozygous and heterozygous P628Qfs*11 mutation in the proband and the brother, respectively. The proband family tree and sequence electropherograms were illustrated in Fig. 2.

Given that the 30 μg iodide/day supplementation partially reversed hypothyroidism in *Slc26a7*-null male mice [8], we suggested to the patient a treatment trial with 1 mg iodide/day after LT4 suspension (TSH 5,72 mIU/L; FT3 5,25 ng/L; FT4 1,02 ng/dL). However, after 13 days, we ceased this treatment and reintroduced LT4 due to the decline of thyroid hormonal profile and the onset of an acute myopericarditis (TSH 17,4 mIU/L; FT4 0,65 ng/dL).

## Discussion and conclusions

We report the case of a 19-year-old man with late-onset dyshormonogenic goiter and hypothyroidism. Thyroid ultrasound revealed diffuse goiter, tracer thyroid uptake was elevated, and the perchlorate-induced iodide discharge test showed a partial organification defect. NGS analysis detected a novel homozygous mutation (P628Qfs*11) in exon 18 of the *SLC26A7* gene, resulting in a truncated protein. In particular, the P628Qfs*11 mutation determines a stop codon at the same position of F631Lfs*8 mutation described by Cangul et al. [8] that, by homology modeling, was predicted to destabilize the carboxyterminal sulfate transporter and anti-sigma factor antagonists (STAS) domain, which is required for membrane localization and exchanger function. The P628Qfs*11 mutant is predicted to remain trapped inside the cells as the F631Lfs*8 mutant, being not able to migrate on the cell membrane.

The first *SLC26A7* gene mutations were described in 2018, identified through NGS in Arabian patients affected by dyshormonogenic CH [10]. A study by Cangul et al. examined 6 families [8], in which at least one case of CH due to *SLC26A7* homozygous mutations was present. All 13 homozygous carriers of *SLC26A7* mutations had CH, with goiter in 8 cases. Heterozygous carriers were euthyroid. Three of these families harbor the F631Lfs*8 mutation and CH was recognized by neonatal screening (NS) in homozygous subjects for the variant. In our case NS data were not available, but the absence of intellectual retardation and the normal growth suggest late onset hypothyroidism. Nevertheless, we can not exclude an early initiation of LT4 replacement therapy for CH, which was then interrupted and denied by the patient.

Similarly, two Japanese siblings with goitrous CH were reported to have a homozygous nonsense mutation in the *SLC26A7* gene (p.Gln500Ter) [9]. In the 15-day-old male neonate CH was diagnosed by NS, while his younger sister was diagnosed with hypothyroidism and goiter at 5 years old. These cases, along with ours, suggest that the same genotype may be associated with phenotypic heterogeneity.

SLCA26A7 null mice exhibit goitrous hypothyroidism but differ from humans in radioiodine studies, showing reduced tracer uptake but normal discharge after perchlorate administration [8]. Cangul et al. [8] demonstrated that 30 µg iodide/day supplementation partially reversed hypothyroidism in *Slc26a7*-null mice. However, the effect of iodine supplementation in patients with *SLC26A7* homozygous mutations remains unexplored. Our trial of 1 mg/die of iodine supplementation for 2 weeks after LT4 suspension did not correct hypothyroidism, but it was interrupted due to concomitant myopericarditis.

Given that Slc26a7-null mice also exhibit distal renal tubular acidosis [8], we investigated acid–base status and urinary electrolytes in our patient, finding normal renal function.

In conclusion, while from literature all CH patients with homozygous mutations of SLC26A7 gene were detected by neonatal screening or within the first years of life, we described the first case of CH due to a homozygous mutation of SLC26A7 diagnosed during late adolescence. Although the NS test was unavailable, the absence of intellectual retardation and the normal growth suggested a late onset of hypothyroidism. We suppose that other environmental factors and genetic polymorphisms of other genes involved in thyroid hormone synthesis may influence iodine transport into the lumen of the thyroid follicles and have a role in the timing and severity of hypothyroidism. No overt renal acid–base abnormalities in the healthy state were observed, but a role of the mutation when homeostasis is perturbed is possible.

## Data Availability

All data generated or analysed during this study are included in this published article.

## Abbreviations

CH: Congenital hypothyroidism
NGS: Next generation sequencing
FT4: Free thyroxine
FT3: Free triiodothyronine
rT3: Reverse free triiodothyronine
TSH: Thyroid stimulating hormone
Tg: Thyroglobulin
anti-Tg antibodies: Anti-thyroglobulin antibodies
anti-TPO antibodies: Anti-thyroid peroxidase antibodies
n.v.: normal values
LT4: L-thyroxine
STAS: Anti-sigma factor antagonists
NS: Neonatal screening
